# Ion dynamics during seizures

**DOI:** 10.3389/fncel.2015.00419

**Published:** 2015-10-21

**Authors:** Joseph V. Raimondo, Richard J. Burman, Arieh A. Katz, Colin J. Akerman

**Affiliations:** ^1^Department of Human Biology, Faculty of Health Sciences, University of Cape TownCape Town, South Africa; ^2^UCT/MRC Receptor Biology Unit, Department of Integrative Biomedical Sciences and Institute of Infectious Disease and Molecular Medicine, Faculty of Health Sciences, University of Cape TownCape Town, South Africa; ^3^Department of Pharmacology, University of OxfordOxford, UK

**Keywords:** epilepsy, seizures, ion dynamics, potassium, sodium, calcium, chloride, pH

## Abstract

Changes in membrane voltage brought about by ion fluxes through voltage and transmitter-gated channels represent the basis of neural activity. As such, electrochemical gradients across the membrane determine the direction and driving force for the flow of ions and are therefore crucial in setting the properties of synaptic transmission and signal propagation. Ion concentration gradients are established by a variety of mechanisms, including specialized transporter proteins. However, transmembrane gradients can be affected by ionic fluxes through channels during periods of elevated neural activity, which in turn are predicted to influence the properties of on-going synaptic transmission. Such activity-induced changes to ion concentration gradients are a feature of both physiological and pathological neural processes. An epileptic seizure is an example of severely perturbed neural activity, which is accompanied by pronounced changes in intracellular and extracellular ion concentrations. Appreciating the factors that contribute to these ion dynamics is critical if we are to understand how a seizure event evolves and is sustained and terminated by neural tissue. Indeed, this issue is of significant clinical importance as status epilepticus—a type of seizure that does not stop of its own accord—is a life-threatening medical emergency. In this review we explore how the transmembrane concentration gradient of the six major ions (K^+^, Na^+^, Cl^−^, Ca^2+^, H^+^and ${\text{HCO}}_{3}^{-}$) is altered during an epileptic seizure. We will first examine each ion individually, before describing how multiple interacting mechanisms between ions might contribute to concentration changes and whether these act to prolong or terminate epileptic activity. In doing so, we will consider how the availability of experimental techniques has both advanced and restricted our ability to study these phenomena.

## Introduction

Epilepsy is a common, debilitating neurological condition which is characterized by recurrent seizures. These striking events often involve severe disruption of brain function due to increased excitation and synchronization in underlying brain networks. In this review we focus on how seizure-associated changes in transmembrane ion concentration gradients might affect on-going pathological activity within brain networks. We explore how the concentration gradients of six major ions (Na^+^, K^+^, Cl^−^, Ca^2+^, H^+^ and ${\text{HCO}}_{3}^{-}$) are regulated under baseline conditions, and how these homeostatic mechanisms may be temporarily overwhelmed in the face of intense ionic fluxes during epileptic seizures.

The term “epileptic seizure” refers to a diverse array of epileptic phenomena from short focal events to generalized tonic-clonic seizures (McCormick and Contreras, 2001). As a result, different seizure activity patterns will be associated with significant variations in the extent and dynamics of the underlying ionic shifts. Brain area and cell type differences contribute to further heterogeneity in ionic responses. In this review we aim to provide a “refresher” on key concepts in the field by integrating findings across different seizure models, neuronal cell types and brain areas to make general points about ion fluxes associated with seizure activity. Unfortunately the format and scope of this review preclude a complete analysis of all the pertinent details associated with this large topic. As we explore ion dynamics during seizure activity, we identify how experimental technologies have either enabled or limited the study of seizure-associated ion dynamics. In our review, we treat each ion in isolation before examining how multiple interacting mechanisms between ions might contribute to concentration changes and whether these act to sustain or terminate epileptic activity. Finally, we discuss how different cell types and subcellular compartments might experience varying seizure-induced changes in ion concentration gradients.

## Ion Concentrations at Rest

Electrochemical signaling in the brain is mediated by ions moving into or out of cells according to their transmembrane electrochemical gradient. The ionic current generated by opening a membrane conductance for a particular ion is proportional to the difference between the membrane potential at that point in time and the reversal potential for the ion in question (this is termed the ionic driving force). The ionic reversal potential in turn is a function of the transmembrane concentration gradient. Ionic concentration gradients are established by both active and passive mechanisms. Impermeable ion species, particularly impermeable intracellular anions such as proteins, result in an unequal distribution of permeable anions and cations across the cell membrane even in the absence of active ion regulation (the Gibbs-Donnan effect). Further to this, the bulk of cerebral metabolism and energy utilization are spent on active mechanisms, which establish the final transmembrane electrochemical ion gradients that sub serve synaptic transmission and action potential generation. The central player in this process is the Na^+^ and K^+^ ATPase active transporter (Na^+^/K^+^ ATPase), which utilizes ATP to establish opposite transmembrane gradients for Na^+^ and K^+^ (Morth et al., 2007). A multitude of channels then utilize these gradients to modulate the electrical potential across the neuronal membrane (see Table 1). For example, K^+^ leak channels contribute to the resting membrane potential while voltage-gated Na^+^ and K^+^ channels underpin the propagating waves of depolarization and repolarization that constitute action potentials (Goldstein et al., 2001; Wood, 2001; Yellen, 2002).

**Table 1:** **Typical ion concentrations at rest and during an epileptic seizure**.

	Typical rest			Typical peak during seizure		
ion	[ion]_i_	[ion]_e_	*E*_ion_	[ion]_i_	[ion]_e_	*E*_ion_	Reference
K^+^	96 mM^1^	4 mM	−85 mV	94 mM	12 mM^2^	−55 mV	Jiang and Haddad (1991) and Dreier and Heinemann (1991)
Na^+^	10 mM^3^	145 mM^4^	+71 mV	55 mM^5^	139 mM^4^	+25 mV	Dietzel et al. (1982), Diarra et al. (2001) and Rose and Konnerth (2001)
Ca^2+^	70 nM	2 mM	+137 mV	700 nM^6^	100 μM^7^	+66 mV	Pumain et al. (1985) and Pal et al. (1999)
Cl^−^	7 mM	145 mM	−80 mV	26 mM^8^	152 mM^4^	−47 mV	Raimondo et al. (2013) and Ellender et al. (2014)
pH/${\text{HCO}}_{3}^{-}$	7.2/15 mM	7.4/24 mM	−13 mV/−13 mV	7.05^9^/10 mM	7.405^10^/25 mM	−25 mV/−25 mV	Caspers and Speckmann (1972) and Raimondo et al. (2012a)
**Receptor**	Relative Permeability		***E*_receptor_**			***E*_receptor_**	
AMPAR	K^+^:Na^+^/1:1		9.1 mV			0.4 mV
GABA_A_R	Cl^−^:${\text{HCO}}_{3}^{-}$/4:1		−70.6 mV			−45.8 mV

*[ion]_i_ and [ion]_e_ indicate the intracellular and extracellular, free ion concentrations, respectively. E_ion_ and E_receptor_ indicate the reversal potentials for ion species and neurotransmitter receptors, respectively. In calculating ${\text{HCO}}_{3}^{-}$ concentrations and E_${\text{HCO}}_{3}^{-}$_ we have assumed that carbon dioxide is equilibrium distributed across the plasma membrane and that the CO_2_ hydration reaction inside and outside the cell is under equilibrium. Values in gray have been estimated where data is not available*.

The Na^+^ and K^+^ gradients generated by the Na^+^/K^+^ ATPase also provide the predominant energy substrate for secondary transport of the other ions addressed in this review. For example, in the mature nervous system the intracellular Cl^−^ concentration is kept at lower levels than would be predicted by passive distribution at a given membrane potential due to the action of the cation-chloride cotransporter KCC2. This transporter uses the K^+^ gradient as the energy source for Cl^−^ extrusion (Kaila et al., 2014a). Interestingly, the transmembrane gradient of Cl^−^ is modulated as a function of development. NKCC1, an alternative cation-chloride cotransporter, which harnesses the Na^+^ gradient to move Cl^−^ intracellularly, plays a more prominent role in setting the intracellular Cl^−^ concentration gradient in young as opposed to mature tissue (Ben-Ari, 2002; Farrant and Kaila, 2007; Kaila et al., 2014a). Sodium-calcium exchangers (NCXs) employ the Na^+^ gradient to extrude Ca^2+^ (Quednau et al., 2004). However, although these low-affinity high-capacity transporters play an important role in Ca^2+^ extrusion, to achieve the sub 100 nM cytoplasmic Ca^2+^ concentrations typical of neurons, additional high affinity, ATP driven mechanisms, (namely plasma-membrane and sarcoplasmic reticulum associated Ca^2+^-ATPase pumps: PMCA, SERCA) are employed (Berridge et al., 2003). Similarly, in the majority of neurons H^+^ export or ${\text{HCO}}_{3}^{-}$ import relies on the Na^+^ gradient to extrude H^+^ (Na^+^–H^+^ exchangers, NHEs) or import ${\text{HCO}}_{3}^{-}$ (Na^+^–${\text{HCO}}_{3}^{-}$ cotransporters, NBCEs or NDCBEs; Chesler, 2003). The final concentration of ${\text{HCO}}_{3}^{-}$ is then intimately linked to that of H^+^ by the reversible reaction of H_2_O and CO_2_ to ${\text{HCO}}_{3}^{-}$ and H^+^, which is catalyzed by the ubiquitous presence of intracellular and extracellular carbonic anhydrases (Chesler, 2003). Hence, alkaline intracellular environments result in relatively high intracellular ${\text{HCO}}_{3}^{-}$ concentrations and it is interesting that the expression and activity of different carbonic anydrases can also be developmentally regulated (Ruusuvuori et al., 2004).

The transport mechanisms described above are arguably the most critical in determining transmembrane ion concentrations at rest. In Table 1 we provide a summary of typical resting intracellular and extracellular, free, neuronal ionic concentrations as well as computed reversal potentials for the major ion species addressed in this review (K^+^, Na^+^, Cl^−^, Ca^2+^, H^+^ and ${\text{HCO}}_{3}^{-}$). At this stage it may be relevant to point out that in determining the functional effect of ion concentration gradients, it is the free concentration, as opposed to the absolute concentration of the ion, which is important. And the free concentration of an ion can be affected differently between the intracellular and extracellular compartments, as a result of differences in dissociation constants caused by the presence of buffers and organic ions. In addition to resting free ion concentrations, Table 1 also provides values for ion concentrations that could occur during an epileptic seizure. These values are averages that have been derived from different cell-types, brain areas and seizure models. As a result, they are intended to provide only an indication of potential ion concentration dynamics and do not capture the full range of values that are likely to be present in the brain.

## Activity-Dependent Changes in Ion Concentrations and their Relationship to Seizure Dynamics

In this section we will examine how the transmembrane concentration gradients for each of the six major ion species can change during an epileptic seizure (K^+^, Na^+^, Cl^−^, Ca^2+^, H^+^ and ${\text{HCO}}_{3}^{-}$), and how this relates to ongoing seizure dynamics.

### Potassium

Changes in extracellular K^+^ concentration represent one of the earliest and most thoroughly described examples of activity-induced ion dynamics (Fertziger and Ranck, 1970; Fröhlich et al., 2008). The development of K^+^ selective electrodes in the early 1970s (Walker, 1971; Singer and Lux, 1975) enabled measurements of extracellular concentrations of K^+^ ([K^+^]_e_) during both physiological and pathological activity. Although early K^+^ selective electrodes based on liquid ion membrane exchange were also sensitive to various neurotransmitters (Kuramoto and Haber, 1981), the development of liquid membrane electrodes using valinomycin are extremely specific for K^+^. In addition, these electrodes can be made with tip sizes below 2 μm, which prevents the formation of large dead spaces within the tissue and allows for accurate sensing of K^+^ in the interstitial space (Ransom et al., 1987; Kaila et al., 1997). Using K^+^ sensitive elctrodes, Singer and Lux (1975) demonstrated increases in [K^+^]_e_ that accompanied physiological stimuli in visual cortex *in vivo* (Singer and Lux, 1975), whilst fluctuations in [K^+^]_e_ have been shown to be coupled to 1 Hz slow oscillations in ketamine-xylazine anaesthetized cats (Amzica and Steriade, 2000). With regard to pathological activity, *in vivo* seizures and *in vitro* seizure-like activity both result in dynamic increases in [K^+^]_e_, reaching peak levels of between 9 and 12 mM (Pumain et al., 1985; Yaari et al., 1986; Avoli et al., 1987; Dreier and Heinemann, 1991; Gnatkovsky et al., 2008). It is worth noting that as the extracellular space accounts for only a fifth of total brain volume with the rest being intracellular (Syková and Nicholson, 2008), it is possible that estimates of changes to extracellular ionic concentration might be underestimated in experiments performed *in vitro*. This is because extracellular ionic changes occurring *in vitro* can be ameliorated by the large volumes of slice perfusate.

Experiments using K^+^ selective electrodes have demonstrated that baseline [K^+^]_e_ is approximately 25-fold lower than [K^+^]_i_ (typically 4 mM vs. 96 mM, Table 1), which means that even small fluxes of K^+^ into the extracellular space results in relatively large changes to the transmembrane K^+^ gradient and consequently the reversal potential for K^+^. Due to the fact that constitutively activated K^+^ conductances (Lesage, 2003) are the major determinant in setting the resting membrane potential, extracellular K^+^ accumulation and accompanying changes to *E*_K+_ result in significant membrane depolarization. Network activity and epileptic seizures result in K^+^ efflux predominantly via intense and prolonged activation of synaptic conductances, particularly by opening glutamate receptors. In addition, membrane depolarization increases K^+^ flux via both voltage-gated and constitutively active K^+^ channels. The K^+^, Cl^−^ cotransporter KCC2, is also responsible for K^+^ extrusion during intense network activity as intracellular Cl^−^ accumulation provides the thermodynamic gradient for outward K^+^ transport (Viitanen et al., 2010). Extracellular K^+^ accumulation is mitigated by a variety of mechanisms including K^+^ transporters on both neurons and astrocytes (Na^+^/K^+^ ATPase, KCC2, NKCC1) as well as passive uptake mechanisms (inward-rectifying K^+^ channels, Kir) on astrocytes (Kofuji and Newman, 2004; Butt and Kalsi, 2006). What is currently still under considerable debate is whether K^+^ is shunted between sites of high to low [K^+^]_e_ through the glial syncytium, a concept termed spatial buffering (Kofuji and Newman, 2004). It is clear however, that under conditions of enhanced activity such as those associated with a seizure, these mechanisms can be overwhelmed and K^+^ is able to accumulate in the extracellular space. For this reason the “potassium accumulation hypothesis” has long been an attractive, albeit contentious, mechanistic explanation for various aspects of seizure activity (Fröhlich et al., 2008). In this scheme, epileptogenic changes result in enhanced extracellular K^+^ accumulation during physiological activity, which triggers the onset of a seizure and a further increase in the [K^+^]_e_. This results in neurons becoming more depolarized, firing more action potentials and releasing further K^+^ into the extracellular space. This runaway, positive-feedback cycle is then thought to be arrested when depolarization is so severe that voltage-gated Na^+^ channels become inactivated and neurons are unable to fire action potentials, a state referred to as depolarization block. Therefore, in this arrangement, extracellular K^+^ accumulation contributes to the initiation, maintenance and termination of a seizure event.

**Figure 1:**
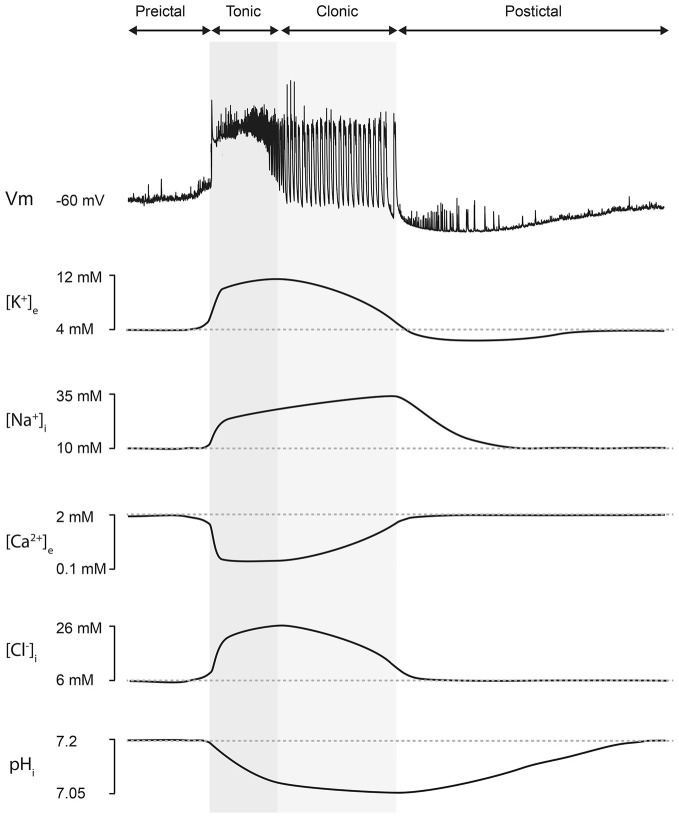
**Ion concentration changes during seizures.** Top, an intracellular recording from a CA3 hippocampal neuron during a seizure-like event *in vitro* demonstrates typical neuronal membrane potential changes which accompany seizure episodes. Seizures consist of a “preictal” phase, a “tonic” phase (characterized by membrane potential depolarization upon which occur high-frequency, low-amplitude discharges, dark gray), a “clonic” or “after-discharge phase” (composed of rhythmic bursts of activity that emerge from a relatively hyperpolarized membrane potential background, light gray) and lastly a “postictal” phase (characterized by a hyperpolarized membrane potential lasting for several minutes before slowly recovering to preseizure values). Bottom, representative changes in ion concentration associated with seizures. The accompanying traces should be viewed as “best approximations” based on the available literature. [K^+^]_e_ is thought to peak at the end of the tonic phase with an undershoot accompanying the postictal period (Dreier and Heinemann, 1991; Fröhlich et al., 2008). The [Na^+^]_i_ trace is inferred from experimental measurements and supported by computational modeling work, which suggests that [Na^+^]_i_ is likely to peak at the end of the seizure (Krishnan and Bazhenov, 2011). [Ca^2+^]_e_ has been shown to drop rapidly to approximately 100 μM during an ictal event (Pumain et al., 1985). [Cl^−^]_i_ increases during seizures and is highest during the clonic phase (Raimondo et al., 2013; Ellender et al., 2014). Neuronal pH_i_ decreases during the seizure, reaching its minimum around the time of seizure offset, and then slowly recovering during the postictal phase (Xiong et al., 2000; Raimondo et al., 2012a).

Although the [K^+^]_e_ has been observed to increase during seizures, considerable uncertainty still exists as to whether this is a cause, rather than simply an effect of epileptiform activity. Indeed, many aspects of the potassium accumulation hypothesis have remained contentious. Firstly, there has been little experimental verification for a [K^+^]_e_ threshold for seizure initiation. Secondly, if the potassium accumulation hypothesis were correct, one would expect that K^+^ would increase during seizure activity and reach a peak as the seizure terminates. In contrast, K^+^ sensitive microelectrode recordings *in vivo* and *in vitro* appear to show that during seizure activity, [K^+^]_e_ peaks mid seizure, that is, at the end of the tonic phase, before decreasing during the clonic phase (Sypert and Ward, 1974; Dreier and Heinemann, 1991; see Figure 1). Similarly, depolarization block of neurons and a cessation of action potential firing does not appear to occur in tandem with seizure termination (Sypert and Ward, 1974). Although, in at least some cases, seizure cessation has been observed to be associated with the onset of spreading depression, depolarization block and a dramatic increase in [K^+^]_e_ (Bragin et al., 1997). For these reasons, the potassium accumulation hypothesis has perhaps been viewed as an oversimplification in terms of accounting for seizure termination. However, some of the general conceptual principles involved in the hypothesis are valid. For example, it is likely that any process that results in increasing levels of depolarization may ultimately become inhibitory by causing depolarization block. Furthermore, considerable experimental and computational data has demonstrated the importance of K^+^ dynamics in the evolution of epileptic seizures. For example, it is well known that raising the extracellular K^+^ concentration of *in vitro* preparations, particularly to the 7.5–8.5 mM range, results in epileptiform activity which can closely resemble seizures with distinct tonic and clonic stages (Traynelis and Dingledine, 1988; Jensen et al., 2014). Importantly, recent computational models incorporating K^+^ ion dynamics have demonstrated that changes to [K^+^]_e_ can explain the transition between tonic and clonic phases of seizures. Indeed, as is observed experimentally, increases in [K^+^]_e_ beyond a certain level can result in the network shifting from a tonic to burst firing mode (Fröhlich et al., 2006, 2010). During the tonic firing phase, extracellular K^+^ once again decreases. In this scheme, extracellular K^+^ still plays an important role in controling the length of epileptiform activity, albeit in a more subtle fashion.

In summary, the relationship between K^+^ dynamics and neural activity is clearly a complex one. Understanding the precise role of K^+^ in seizure maintenance and termination will no doubt require combining such theoretical work with more sophisticated means of experimentally measuring changes in K^+^ concentration. This might include the further development of K^+^ sensitive dyes (Rimmele and Chatton, 2014) and genetically-encoded K^+^ sensors, akin to those available for Ca^2+^, Cl^−^ and pH.

### Sodium

The potential for, and relevance of, changes to the transmembrane concentration gradient of Na^+^ during epileptic seizures has not been extensively investigated. This is largely due to the technical difficulties associated with recording the concentration of this ion within nervous tissue. Although Na^+^ sensitive electrodes have existed for several decades, their size has largely limited their use to extracellular measurements. In addition, the high baseline extracellular concentration of Na^+^ ([Na^+^]_e_) reduces the signal to noise of recorded changes in extracellular Na^+^ concentration. That said, the data available indicates that epileptiform activity is associated with a peak reduction in [Na^+^]_e_ of approximately 4–7 mM (a typical resting [Na^+^]_e_ is 145 mM, see Table 1; Dietzel et al., 1982). This measurement was made without volume correction, which is important to note as the volume of the extracellular space has been reported to contract by approximately 30% during seizures (Dietzel and Heinemann, 1986). Without this volume contraction and its “concentrating effect”, the reduction in [Na^+^]_e_ would be expected to be greater.

Decreases in extracellular Na^+^ occur in tandem with an increase in the concentration of intracellular Na^+^. The development of Na^+^ sensitive dyes, the most popular being the ratiometric Na^+^ indicator SBFI (sodium binding benzofuran isophthalate) allows the quantitative measurement of [Na^+^]_i_ utilising epifluorescence or more effectively, 2-photon microscopy (Minta and Tsien, 1989; Rose, 2003; Meier et al., 2006). A number of groups have used SBFI to quantify intracellular Na^+^ accumulation during synaptically-evoked and epileptiform activity (Rose and Konnerth, 2001; Langer and Rose, 2009; Fleidervish et al., 2010; Karus et al., 2015). This influx of Na^+^ appears to be predominantly mediated by dendritic NMDA receptors (NMDARs), although Na^+^ fluxes via other Na^+^ conductances, particularly voltage-gated Na^+^ channels, are also expected to contribute (Rose and Konnerth, 2001; Langer and Rose, 2009). It is therefore likely that significant increases in [Na^+^]_i_ follow the intense neural activity and activation of NMDARs that accompanies epileptic seizures. This [Na^+^]_i_ increase is likely to be most pronounced within dendritic spines as glutamatergic synapses on the spine head are the site of predominant influx, and the small, restricted volume of the spine will result in enhanced concentration increases for a given flux of Na^+^ (Rose and Konnerth, 2001). However, the exact temporal and spatial quantification of these seizure-associated Na^+^ fluxes remain to be shown.

It is expected that a progressive breakdown in the transmembrane Na^+^ gradient should serve to reduce neural excitability and network activity. Inhibitory effects following seizure-associated changes in the Na^+^ gradient are likely to occur via a number of possible mechanisms. One such mechanism is through a reduction in the driving force for voltage gated Na^+^ channels. This would alter action potential kinetics and could reduce the ability of neighboring voltage-gated Na^+^ channels to recruit each other during the course of action potential generation. Based on a peak intracellular concentration of 55 mM and an extracellular concentration of 139 mM, it is estimated that the Na^+^ reversal would shift from +71 mV at rest to a minimum of +25 mV during a seizure (Dietzel et al., 1982; Rose and Konnerth, 2001). Similarly, intracellular accumulations of Na^+^ would reduce the Na^+^ driving force at glutamatergic receptors, reducing the size of excitatory postsynaptic currents. However, as glutamate receptors are equally permable to K^+^, this effect is somewhat counterbalanced by simultaneous reductions in the driving for K^+^ (see *E*_AMPA_ in Table 1). Computational modeling, which accounts for the dynamics of multiple ions in the context of epileptiform activity, has shown that the progressive intracellular increase of Na^+^ is likely to be an important factor in triggering the termination of seizure activity. Compatible with changes in Na^+^ altering excitability, in these simulations intracellular Na^+^ peaks immediately prior to seizure offset (Krishnan and Bazhenov, 2011; see Figure 1).

Another mechanism by which increases in [Na^+^]_i_ might have an inhibitory effect is via activation of the electrogenic Na^+^/K^+^ ATPase. Repetitive action-potential generation and increases in Na^+^ are well known to stimulate the activity of this transporter (Ritchie and Straub, 1957; McDougal and Osborn, 1976; Thompson and Prince, 1986). Due to the fact that the transporter pumps two K^+^ ions into the cell for every three Na^+^ ions extruded, enhanced activity will have a hyperpolarizing effect on the neuronal membrane potential (Krishnan and Bazhenov, 2011; Krishnan et al., 2015). Lastly, the relatively recent discovery of the Na^+^ -activated K^+^ family of channels, “Slick” and “Slack” offer a further important mechanism by which increases in [Na^+^]_i_ could terminate epileptic seizures (Igelstrom, 2013). These channels, which are activated by intracellular Na^+^ accumulation, have been shown to result in powerful and sustained membrane hyperpolarization following periods of spiking activity (Bhattacharjee et al., 2003; Yuan et al., 2003). As such, Slick and Slack are well placed to play a role in the spontaneous termination of epileptic seizures (Igelstrom, 2013).

Finally, indirect evidence supporting the role of Na^+^ dynamics in seizure cessation comes from data on the effects of common antiepileptic drugs that target Na^+^ channels. For example, the anticonvulsant effect of phenytoin is linked to its inhibitory action on voltage-gated Na^+^ channels. Whilst this anticonvulsant agent effectively raises the threshold for seizure generation, it has also been shown to extend the duration of seizures (Ebert et al., 1997). It is possible that this effect is due to slower seizure-associated intracellular Na^+^ accumulation on account of reduced voltage-gated Na^+^ currents.

### Calcium

Under typical baseline conditions the free Ca^2+^ concentration within neurons is below 100 nM. As a result, activity-dependent Ca^2+^ influx causes large changes in free [Ca^2+^]_i_, constituting a powerful and reliable intracellular signal (Collingridge and Bliss, 1987; Collins et al., 1991; Franklin and Johnson, 1992; Ghosh and Greenberg, 1995). Due to the involvement of Ca^2+^ in so many fundamental cellular processes, and the large relative changes in its concentration, considerable attention has been devoted to developing tools for monitoring changes in [Ca^2+^]_i_. Indeed, observing changes in [Ca^2+^]_i_ is commonly and reliably used as a proxy for neural activity. Although Ca^2+^ sensitive electrodes do exist, the small molecule Ca^2+^ dyes (indicators like Oregon Green BAPTA-1, fura-2 and fluo-4) have until recently been the workhorses of intracellular Ca^2+^ measurement. These indicators are now gradually being superseded by genetically-encoded Ca^2+^ reporters, of which the GCaMP family of proteins is the most popular. Targeted mutagenesis has resulted in iterative functional improvements in these reporters, which now exhibit kinetic properties approaching those of the Ca^2+^ dyes. The major advantage of these sensors is that they can be genetically targeted to specific cell-populations or subcellular locations (Looger and Griesbeck, 2012; Chen et al., 2013). An important caveat however, is that the majority of the Ca^2+^ reporters described above are non-ratiometric and, where ratiometric reporters have been used in epilepsy studies (e.g., Fura-2 and Twitch), these have rarely been used in their calibrated form. This means that recorded Ca^2+^ signals are qualitative and provide a read out of neuronal activity, rather than quantitative measurements of Ca^2+^ concentration. In addition, many of the reporters are ultimately limited in their ability to resolve subcellular Ca^2+^ signaling processes, which can occur in microdomains close to sites of Ca^2+^ influx. Therefore studies to date have tended to focus on the more widespread increases in intracellular Ca^2+^ that are observed during seizure-activity.

During the course of an epileptic seizure, Ca^2+^ influx occurs predominantly via voltage-gated Ca^2+^ channels and NMDARs. Membrane depolarization simultaneously activates the voltage-gated channels and releases the voltage-dependent Mg^2+^ block of the NMDAR. This combines with high levels of glutamate released during the seizure to generate a pronounced Ca^2+^ influx and rapid accumulation of intracellular Ca^2+^. As is the case for Na^+^, seizure-associated increases in [Ca^2+^]_i_ are likely to be very large within dendritic spines due to their small volume and the strong activation of NMDARs at spine-associated glutamatergic synapses. Calcium imaging during the course of seizure activity demonstrates that Ca^2+^ influx occurs in almost all neurons and astrocytes during an ictal event (Trevelyan et al., 2007). As described above, the vast majority of studies have used relative, as opposed to absolute reporters of Ca^2+^. This means that surprisingly little quantitative data exists describing Ca^2+^ concentration changes during seizures. However, the data that does exist suggests that the extracellular concentration of Ca^2+^ drops significantly in tandem with the rapid and widespread flow of Ca^2+^ into cells during an ictal event (Benninger et al., 1980; Somjen, 1980; Pumain et al., 1985; Pal et al., 1999; also see Figure 1). Indeed, *in vivo* measurements of extracellular Ca^2+^ concentration in primates during seizures has shown that Ca^2+^ drops to within the 100 μM range (Pumain et al., 1985; Figure 1). At this concentration, synaptic transmission is significantly compromized (Feng and Durand, 2003), as there is too little extracellular Ca^2+^ available to sustain the Ca^2+^ influxes necessary for vesicle fusion and synaptic release of neurotransmitter. In addition, the dissipation of the transmembrane Ca^2+^ gradient would cause a negative shift in the reversal potential for Ca^2+^ (see Table 1). Furthermore, Ca^2+^ entry also results in the opening of Ca^2+^-activated K^+^ conductances (Vergara, 1998). One would predict all of these processes to play a negative feedback or inhibitory role, and thereby serve to terminate a seizure.

Despite this prediction, it is also known that the experimental removal of Ca^2+^ from the extracellular space reliably evokes seizure-like activity both *in vitro* and *in vivo* (Haas and Jefferys, 1984; Feng and Durand, 2003). Indeed, reduced extracellular Ca^2+^ is an established model of non-synaptic epileptiform activity. The reduction in extracellular Ca^2+^ is believed to reduce surface charge screening, which shifts the conductance-voltage relationship of voltage-gated channels. Voltage-gated channels are therefore more likely to be activated and thus cause enhanced excitability (McLaughlin et al., 1971). Furthermore, with the desynchronising effect of synaptic noise removed, local field effects are able to result in ephaptic coupling and synchronization of neurons (Taylor and Dudek, 1982). These phenomena are particularly prevalent in the rodent hippocampus where the somata of pyramidal cells are tightly packed, although this aspect of hippocampal anatomy is much less pronounced in the human (West and Gundersen, 1990). Rodent studies have shown that experimental manipulation of extracellular osmolality, which in turn effects cell swelling, the size of the extracellular space and hence ephaptic coupling, directly controls the duration of zero Ca^2+^ induced epileptiform bursts (Dudek et al., 1990). It has been suggested that zero Ca^2+^ induced epileptiform activity may represent a useful analog of the ictal phase of epileptic seizures. The reduced extracellular Ca^2+^ concentration and cell swelling associated with seizures (Olsson et al., 2006) mean that the non-synaptic mechanisms contributing to zero Ca^2+^ epileptiform activity, could play an important role in extending the ictal phase of epileptic seizures.

### Chloride

The transmembrane Cl^−^ gradient has a critical influence on network activity due to the fact that the channels responsible for fast synaptic inhibition, GABA_A_ receptors (GABA_A_Rs), are primarily permeable to Cl^−^. Open GABA_A_Rs are approximately 4 times more permeable to Cl^−^ than to ${\text{HCO}}_{3}^{-}$ (Kaila and Voipio, 1987). Therefore at rest, *E*_GABAA_ (typically around −70 mV) is much closer to the very negative Cl^−^ reversal (*E*_Cl^−^_; typically −85 mV) than the considerably more positive ${\text{HCO}}_{3}^{-}$ reversal (*E*_${\text{HCO}}_{3}^{-}$_; typically −13 mV; Bormann et al., 1987; Kaila et al., 1993). *E*_GABAA′s_ proximity to the neuronal resting membrane potential means that relatively small changes in [Cl^−^]_i_, and hence *E*_GABAA_ can greatly influence the functional effect of GABA_A_R activation and consequently neuronal output.

Inhibitory synapses are particularly prone to short-term activity-dependent changes in [Cl^−^]_i_. Indeed, Cl^−^ accumulation is often used as the definitive example of what is referred to as short-term ionic plasticity (Rivera et al., 2004; Raimondo et al., 2012b; Kaila et al., 2014b). This is any process that results in short-lasting changes to the ionic driving force for post-synaptic receptors. The study of Cl^−^ dynamics in the context of network activity has been aided by experimental tools which are either able to determine [Cl^−^]_i_ indirectly, via the measurement of *E*_GABAA_ using the gramicidin perforated patch clamp technique (Kyrozis and Reichling, 1995) or single channel recordings in cell attached mode (Tyzio et al., 2008), or directly by using fluorescent Cl^−^ reporters (Kuner and Augustine, 2000; Markova et al., 2008; Arosio et al., 2010; Raimondo et al., 2013). It should be noted that genetically encoded Cl^−^ indicators, whilst enabling subcellular and cell-type specific targeting, suffer from intrinsic pH sensitivity. This is especially problematic in the context of measuring Cl^−^ fluxes during seizures, which are also expected to generate significant pH shifts (Raimondo et al., 2012a). However, the latest Cl^−^ reporters based on a novel fusion protein (Arosio et al., 2010; Raimondo et al., 2013) afford simultaneous measurements of pH, which allows the Cl^−^ signal to be corrected for concurrent shifts in pH. Although this is a relatively complex correction procedure with the potential for measurement artifacts to occur if performed incorrectly. Experiments using these techniques have definitively demonstrated that intense activation of GABA_A_Rs, particularly in tandem with concurrent membrane depolarization, can overwhelm endogenous Cl^−^ extrusion systems such as the cation-chloride cotransporter KCC2 and result in rapid intracellular Cl^−^ accumulation (Kaila and Voipio, 1987; Kaila et al., 1989; Thompson and Gähwiler, 1989; Staley et al., 1995; Staley and Proctor, 1999; Ellender et al., 2014). Intracellular Cl^−^ accumulation during intense activation of GABA_A_Rs can occur even in the absence of concurrent depolarization mediated by other conductances. This is because GABA_A_Rs are also permeable to ${\text{HCO}}_{3}^{-}$. A corresponding collapse of the ${\text{HCO}}_{3}^{-}$ gradient is prevented by the activity of intra- and extracellular carbonic anhydrases, which use CO_2_ as a substrate to rapidly regenerate intracellular ${\text{HCO}}_{3}^{-}$ (Kaila et al., 1990; Rivera et al., 2005). As a result, the relatively stable and more positive *E*_${\text{HCO}}_{3}^{-}$_ maintains the driving force for continued intracellular Cl^−^ accumulation during repeated activation of GABA_A_Rs. Interestingly this process appears to be developmentally controled, at least in rats, where the appropriate isoforms of carbonic anhydrase are up regulated at postnatal day 12 (Ruusuvuori et al., 2004). Intracellular Cl^−^ accumulation not only reduces the size of subsequent GABA_A_R mediated hyperpolarizing potentials, but can ultimately render GABAergic transmission depolarising or even excitatory. Epileptic seizures are characterized by widespread membrane depolarization and powerful activation of neurotransmitter receptors including GABA_A_Rs. It is therefore unsurprising that significant Cl^−^ accumulation has been shown to accompany epileptiform activity in a wide array of *in vitro* seizure models (Lamsa and Kaila, 1997; Isomura et al., 2003; Fujiwara-Tsukamoto et al., 2010; Ilie et al., 2012; Raimondo et al., 2013; Ellender et al., 2014). Gramicidin perforated patch recordings indicate seizure-associated depolarising shifts in *E*_GABAA_ in the region of 30 mV (Ellender et al., 2014). Similarly, dynamic readout of [Cl^−^]_i_ using genetically encoded Cl^−^ sensors have observed seizure induced Cl^−^ accumulation between 10 and 20 mM (Raimondo et al., 2013). Ion sensitive microelectrode recordings of [Cl^−^]_e_ have demonstrated that although Cl^−^ moves into the intracellular space, [Cl^−^]_e_ actually increases by about 7 mM during seizures. This is because the extracellular space contracts by about 30% “concentrating” the remaining extracellular Cl^−^. Nonetheless, these changes to the electrochemical gradient for Cl^−^ result in GABAergic transmission being rendered excitatory and hence serves to exacerbate rather than abort epileptiform activity (see Table 1 and Figure 1). Indeed, computational models have suggested that the extent of intracellular Cl^−^ accumulation is a pivotal determinant of seizure duration (Krishnan and Bazhenov, 2011). Experimental evidence has confirmed that excitatory GABAergic signaling maintains the clonic phase of seizure-like events *in vitro* (Fujiwara-Tsukamoto et al., 2010; Ellender et al., 2014). Although Cl^−^ accumulation and seizure-induced excitatory shifts in GABAergic signaling remain to be demonstrated *in vivo*, it is likely that Cl^−^ dynamics play a role in determining when seizures terminate.

### Hydrogen and Bicarbonate

**Figure 2:**
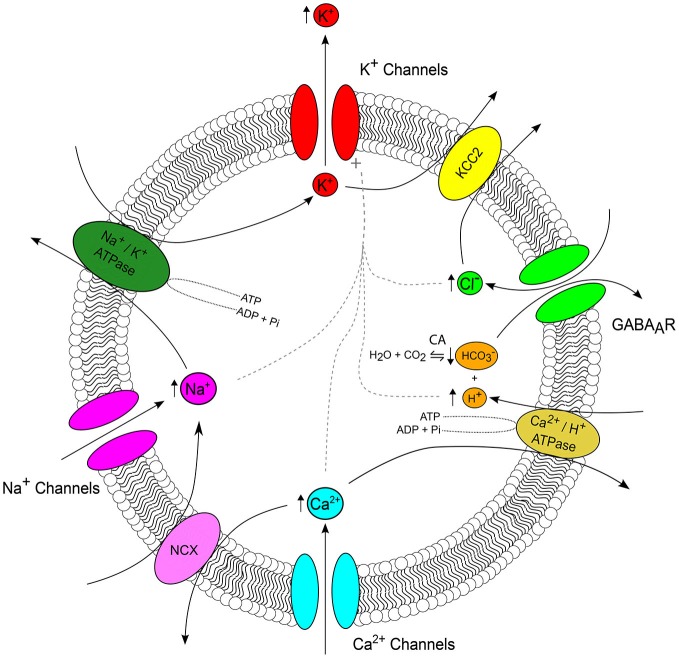
**Ion interactions during seizures.** The major ion channels and transporters, which serve as important nodes of interaction between ions are depicted, with arrows highlighting the typical direction of ionic flux. Arrows adjacent to an ion reflect the direction of seizure induced concentration change (see Figure 1). During a seizure, K^+^ efflux via a host of K^+^ channels (red) results in accumulation of extracellular K^+^. The cation-chloride transporter KCC2 (yellow) serves as an important link between the seizure-associated reduction in the transmembrane K^+^ gradient and intracellular Cl^−^ accumulation via GABAA receptors (GABA_A_Rs) (green). GABA_A_Rs, which are permeable to both Cl^−^ and ${\text{HCO}}_{3}^{-}$ connect the regulation of Cl^−^, ${\text{HCO}}_{3}^{-}$ and pH via carbonic anhydrases which catalyze the reversible reaction of H_2_O and CO_2_ to ${\text{HCO}}_{3}^{-}$ and H^+^ (orange). Seizures are associated with intracellular acidification which is due, in part, to the activity of Ca^2+^/H^+^ ATPase as it imports H^+^ and extrudes Ca^2+^ in attempt to restore baseline Ca^2+^ concentrations following activity-induced Ca^2+^ influx (cyan). Na^+^/Ca^2+^ exchangers (NCX, pink) connect Ca^2+^ and Na^+^ concentration. Seizure-associated Na^+^ influx via voltage and ligand gated Na^+^ channels (magenta) up regulates the activity of Na^+^/K^+^ ATPases (forest green). Finally, increased intra-neuronal concentrations of Cl^−^, H^+^, Ca^2+^ and Na^+^ all activate K^+^ channels (dashed gray line). The number and complexity of possible ionic interactions highlights the importance of computational models for determining the relevance of these continuously evolving variables, which are often difficult to study experimentally.

Due to the effect of pH on protein folding and enzymatic activity, it is a critical parameter that is tightly regulated for optimum cell function. pH is fundamental to a variety of cellular processes including cell division, metabolism, apoptosis and migration (Opie, 1965; Denker and Barber, 2002; Putney and Barber, 2003; Abad et al., 2004). Within the nervous system, the control of pH has particular relevance for synaptic transmission and the modulation of network excitability (Drapeau and Nachshen, 1988; Tabb et al., 1992; Dulla et al., 2005). In general, alkalosis increases neuronal excitability whilst acidosis reduces neural excitability (Balestrino and Somjen, 1988; Sparing et al., 2007; Tolner et al., 2011). Multiple endogenous buffer and transport systems exist to control both intracellular and extracellular pH. One of the most important is the ${\text{HCO}}_{3}^{-}$ buffer system, which utilizes intra- and extracellular carbonic anhydrases to catalyze the reversible reaction of H_2_O and CO_2_ to ${\text{HCO}}_{3}^{-}$ and H^+^. As a result, pH and ${\text{HCO}}_{3}^{-}$ concentration are closely linked (see Figure 2).

Multiple experimental tools exist for the measurement of pH including pH-sensitive micro-electrodes, pH-sensitive fluorescent dyes and genetically encoded pH sensors (Rose and Deitmer, 1995; Boyarsky et al., 1988; Buckler and Vaughan-Jones, 1990; Kneen et al., 1998). Despite the presence of intrinsic buffering systems, the use of these tools has demonstrated a plethora of activity-dependent pH changes in multiple systems (Chesler, 2003). Measurements of intraneuronal pH in the context of *in vitro* seizure models has revealed intracellular acidification which increases as a function of seizure duration (Xiong et al., 2000; Raimondo et al., 2012a). For seizure-like events longer that 30 s, peak shifts often exceeded 0.2 pH units and are closely correlated with the termination of epileptiform activity (Xiong et al., 2000; Raimondo et al., 2012a; Figure 1). At least three major processes are likely to be involved in seizure-induced acidification. Firstly, a fall in pH is linked to the activity-induced entry of Ca^2+^ which then requires extrusion via the function of Ca^2+^/H^+^ ATPases located in the plasma membrane and endoplasmic reticulum (Schwiening et al., 1993; Makani and Chesler, 2010). Secondly, prolonged neural activity will increase the production of metabolic acids such as CO_2_ and lactate (Wang et al., 1994). And thirdly, the intense GABA_A_R activation that accompanies seizure activity has been shown to result in considerable ${\text{HCO}}_{3}^{-}$ efflux and a resulting intracellular acidification (Pasternack et al., 1993; Trapp et al., 1996). With regard to the extracellular space, seizures or electrically stimulated activity are associated with more complex pH transients, which may be biphasic (an initial extracellular alkaline shift followed by a prolonged acidosis; Caspers and Speckmann, 1972; Urbanics et al., 1978; Xiong and Stringer, 2000). It is likely that this complexity is due to the fact that pH transients observed in the extracellular space must somehow reflect the combined consequence of seizure-associated intracellular pH transients in both neurons and other cell types, such as astrocytes (Chesler, 2003).

Progressive intraneuronal acidification during a seizure has been suggested to be an important factor influencing seizure termination. Although the precise mechanisms remain unknown, it is likely that acidification could alter excitability by modulating the conductance of ligand and voltage-gated channels. For example, acidification has been shown to reduce the conductance of NMDARs (Tang et al., 1990; Traynelis and Cull-Candy, 1990; Vyklický et al., 1990), enhance the conductance of GABA_A_Rs (Takeuchi and Takeuchi, 1967; Smart and Constanti, 1982; Pasternack et al., 1996) and reduce voltage gated Ca^2+^ currents (Iijima et al., 1986; Barnes and Bui, 1991; Tombaugh and Somjen, 1997; Tombaugh, 1998). Furthermore, intracellular acidification is known to reduce gap junction coupling (Spray et al., 1981), which would serve to reduce the neuronal synchronization that accompanies seizures (de Curtis et al., 1998). Indeed, metabolic acidosis, which would lower intracellular pH, could be relevant for the mechanism of action of some antiepileptic treatments including the ketogenic diet (Neal et al., 2008) and carbonic anhydrase inhibitors (Velisek and Veliskova, 1993).

## Interacting Mechanisms that Influence Ion Dynamics and Neurotransmission

The above discussion has highlighted the fact that epileptic seizures are associated with marked changes to the transmembrane concentration gradient for the six major ions (K^+^, Na^+^, Cl^−^, Ca^2+^, H^+^ and ${\text{HCO}}_{3}^{-}$). What adds a significant layer of complexity to this picture, is the fact that these ionic fluxes interact in multiple ways during the evolution of a seizure. In this section we briefly highlight several important nodes of interaction between these ion species (see Figure 2).

Increased K^+^ conductance and K^+^ efflux is a common consequence of the seizure-associated accumulation of Na^+^, Cl^−^, Ca^2+^ and H^+^. Increases in [Na^+^]_i_ and [Ca^2+^]_i_ trigger the activation of specialized families of K^+^ channels, which are gated by either Na^+^ (Igelstrom, 2013) or Ca^2+^ (Vergara, 1998). Similarly, raised intracellular Cl^−^ co-operatively activates both Slick and Slack (Kaczmarek, 2013), and acidic intracellular pH enhances the conductance of inwardly rectifying K^+^ channels (Yang and Jiang, 1999). Enhanced K^+^ efflux, whilst having a hyperpolarising effect on the membrane potential, also results in a dissipation of the transmembrane K^+^ gradient and an accumulation of extracellular K^+^ with complex effects on network excitability as described in the potassium section above.

The cation-chloride transporter KCC2 serves as an important link between dynamic changes in K^+^ and Cl^−^ (Figure 2). As a cotransporter, KCC2 relies on the thermodynamic driving force for K^+^ and Cl^−^ to transport both ions in the same direction across the membrane. Therefore, increases in extracellular K^+^ during a seizure are predicted to reduce the ability of KCC2 to utilize the K^+^ gradient to extrude Cl^−^. Viewed from an alternative perspective, the accumulation in intracellular Cl^−^ that is mediated by intense activation of the GABA_A_R during a seizure would be expected to enhance K^+^ extrusion via KCC2 (Viitanen et al., 2010), albeit until extracellular K^+^ accumulation reaches a level that renders outward transport of both ions energetically unfavorable. This example demonstrates how dynamic changes in the concentration of the principle ions can exert complex forms of cross talk.

${\text{HCO}}_{3}^{-}$ ions provide a pivotal node of interaction between neuronal regulation of Cl^−^ and H^+^. This is due to the fact that GABA_A_Rs are permeable to both Cl^−^ and ${\text{HCO}}_{3}^{-}$, and because intracellular and extracellular carbonic anhydrases link pH to [${\text{HCO}}_{3}^{-}$]_i_ via the reversible reaction of H_2_O and CO_2_ to ${\text{HCO}}_{3}^{-}$ and H^+^ (see Figure 2). Although carbonic anhydrases are largely able to regenerate intracellular ${\text{HCO}}_{3}^{-}$ in the face of the ${\text{HCO}}_{3}^{-}$ efflux associated with intense activation of GABA_A_Rs, the generation of intracellular H^+^ that accompanies epileptic activity will shift the equilibrium set point of the catalyzed reaction of H_2_O and CO_2_, to ${\text{HCO}}_{3}^{-}$ and H^+^. Therefore, activity induced acidification is predicted to cause a reduction in intracellular ${\text{HCO}}_{3}^{-}$ and hence a hyperpolarization of *E*_${\text{HCO}}_{3}^{-}$_ (Table 1). As *E*_GABAA_ is a product of both *E*_${\text{HCO}}_{3}^{-}$_ and *E*_Cl^−^_, this progressive acidification should reduce the depolarising shift in *E*_GABAA_, which occurs following seizure induced Cl^−^ accumulation (Doyon et al., 2011).

Finally, the transmembrane transport of neurotransmitters such as glutamate (Excitatory Amino Acid Transporter—EAAT1–5) and GABA (GABA Transporter—GAT1–3) are intimately linked to the electrochemical gradients of several ion species. Glutamate transport in both neurons and astrocytes is driven by the electrochemical gradients for glutamate, Na^+^, H^+^ and K^+^ (Danbolt, 2001). Interestingly, several of the glutamate transporters (EAAT1,3,4) also act as glutamate sensitive Cl^−^ channels (Fairman et al., 1995). Similarly, GABA transport depends on the electrochemical gradients of GABA, Na^+^ and Cl^−^. Whilst the activation of ionotropic receptors for GABA and glutamate, as discussed extensively above, results in alterations to the transmembrane gradient for multiple ion species. These changes to the transmembrane ion gradients in turn affect the direction and rate of neurotransmitter transport across cell membranes. For example, the depolarization and increased intracellular Na^+^ concentrations that occur during seizures are predicted to result in a reversal in the direction of GABA transport by GABA transporters (Wu et al., 2007), switching from reuptake to extrusion of GABA into the extracellular space (During et al., 1995).

These multifaceted interactions between the different ion concentration dynamics, electrochemical gradients and synaptic transmission remind us of the difficulties involved in investigating these phenomena. Indeed, this is perhaps the greatest challenge for the field, and underscores the potential importance of computational models for determining the relevance and impact of continuously interacting and evolving variables, which can be difficult to tease apart experimentally.

## Ion Dynamics in Non-Neuronal Cell Types and Subcellular Compartments

So far we have focussed on ion concentration gradients across the neuronal membrane. It is important to note that astrocytes are interwoven into the fabric of neuronal networks where, like neurons, they express a host of proteins that establish particular ion concentration gradients across their membranes. By affecting ion concentrations within the extracellular space, astrocytes also modulate the ion concentration gradients that are critical for synaptic transmission and network excitability. One of the best described roles for astrocytes entails the control of extracellular K^+^ as described in the potassium section above. Astrocytes act as a K^+^ “sink” that prevents excessive extracellular build-up of K^+^ during neuronal activity. Indeed, in the context of seizure activity, there is accumulating evidence from animal models and tissue excised from patients with temporal lobe epilepsy, that astrocytic dysfunction plays a significant role in epileptogenesis, particularly with regard to extracellular K^+^ regulation (Hinterkeuser et al., 2000; Steinhäuser and Seifert, 2002; Wallraff et al., 2006; Bedner et al., 2015).

Compared to neuronal studies, measurements of intra-astrocytic ion concentration dynamics during epileptiform activity have received much less attention. Nonetheless, elevated network activity is thought to result in increases in intra-astrocytic Ca^2+^, Na^+^ and K^+^, as well as an intra-astrocytic alkalinization (Chesler and Kraig, 1989; Walz, 2000; Volterra et al., 2014; Karus et al., 2015). The direction of activity-induced astrocytic Cl^−^ flux is still quite unclear, although GABA_A_R activation has been suggested to result in Cl^−^ efflux from astrocytes (Egawa et al., 2013). If these changes are also evident during a seizure, astrocytic movement of K^+^ and H^+^ would be expected to ameliorate the seizure-induced changes in the concentration of these ions in neurons. Meanwhile, the astrocytic movement of Ca^2+^ and Na^+^ would be expected to have the opposite effect, and exacerbate the changes in neurons. Determining the relevance of ion dynamics in different cell types for seizure propagation and termination represents an interesting area for future research.

In addition to the cell-type specific control of ion concentration gradients, different subcellular compartments within an individual neuron or astrocyte can also show differences in ion concentration. Such subcellular differences may result from the expression pattern of ion transporters, or the particular influxes of ion species into different regions of a cell. Local concentration changes resulting from ionic fluxes across the membrane are a function of the diffusive and ion transport properties of the compartment concerned, as well as the volume. For example, a given flux of ions will cause a greater concentration change in a small intracellular compartment, compared to a large volume compartment. Indeed, experimental and computational studies have shown that a flux of Cl^−^ will cause a greater change in [Cl^−^]_i_ and hence *E*_GABAA_ when Cl^−^ loading occurs within dendrites, than in the soma (Staley and Proctor, 1999; Raimondo et al., 2012b). As discussed in preceding sections, this is also relevant for Na^+^ and Ca^2+^ influxes associated with activity-induced glutamatergic conductances on dendritic spines. The ionic changes that occur during a seizure are therefore a function of the site of flux and the properties of the compartment concerned. Recent evidence has emerged that, at least within *in vitro* models of seizures, strong recruitment of soma-targeting parvalbumin expressing interneurons means that the site of maximal Cl^−^ accumulation occurs within somatic as opposed to dendritic regions of hippocampal pyramidal neurons (Ellender et al., 2014). However, it is fair to say that subcellular differences in seizure-evoked ionic changes and their functional relevance for on-going pathological activity are poorly described. This is therefore an area of research that should benefit greatly from advances in genetically-encoded ion sensors and related optogenetic techniques.

## Conclusion and Relevance of Ion Dynamics in Epilepsy

Increased knowledge of seizure-associated ion dynamics will no doubt have important implications for anticonvulsant therapies, particularly in the context of treatments for status epilepticus. In the case of status epilepticus, endogenous inhibitory processes are unable to terminate seizure activity and it seems highly likely that perturbed ion concentration gradients play an important role in this pathological state. It is therefore important to avoid the assumption that agents that have a particular effect on synaptic signaling under resting conditions, will have the same effect during status epilepticus. As a potential example, agents from the benzodiazepine class of drugs, which are positive modulators of GABA_A_R signaling, are commonly used as the first line therapy in the treatment of status epilepticus. If seizure mediated intracellular Cl^−^ accumulation, as described above, has rendered fast GABAergic transmission excitatory within major brain areas, it is possible that these agents may not be ideally suited for aborting pathological hyperexcitability (Deeb et al., 2013). Although this remains to be demonstrated experimentally, seizure-associated Cl^−^ accumulation may be part of the explanation as to why benzodiazepines often fail to halt status epilepticus (Yasiry and Shorvon, 2014). In this light, novel therapies that take into account the perturbed ion concentration gradients of a seizure may be more appropriate. For instance, the development of strategies to enhance ion transport and the restoration of ion concentration gradients may constitute a valuable strategy for aborting or preventing extended seizure states (Gagnon et al., 2013).

Seizure termination is difficult to study experimentally as the manipulation of potential terminating mechanisms is likely to involve variables that also influence seizure initiation and propagation. Furthermore, during the peak of the ictal state multiple processes occur simultaneously and influence one another in complex ways. Nonetheless, it is clear that seizure-associated changes in transmembrane ion concentration gradients play an important role in the evolution and continuation of pathological network activity. Future research will no doubt take advantage of novel genetically-targeted ion sensors and powerful computing technologies in order to determine how multiple ion species interact in the context of epileptic seizures. It is our hope that a better appreciation of the potential influence of ion dynamics in seizures will stimulate novel avenues of epilepsy research and ultimately result in improved intervention strategies.

## Conflict of Interest Statement

The authors declare that the research was conducted in the absence of any commercial or financial relationships that could be construed as a potential conflict of interest.
